# Associations between rare microglia-linked Alzheimer's disease risk variants and subcortical brain volumes in young individuals

**DOI:** 10.1016/j.dadm.2019.03.005

**Published:** 2019-05-02

**Authors:** Thomas M. Lancaster

**Affiliations:** a Cardiff University Brain Research Imaging Centre (CUBRIC), School of Psychology, Cardiff University, Cardiff, UK; b UK Dementia Research Institute, School of Medicine, Cardiff University, UK

**Keywords:** Magnetic resonance imaging, Freesurfer, Alzheimer's disease, Microglia, Exome-sequencing, *TREM2*

## Abstract

**Introduction:**

Recent exome sequencing studies have identified three novel risk variants associated with Alzheimer's disease (AD). However, the mechanisms by which these variants confer risk are largely unknown.

**Methods:**

In the present study, the impact of these rare coding variants (in *ABI3, PLCG2*, and *TREM2*) on all subcortical volumes is determined in a large sample of young healthy individuals (N = 756–765; aged 22–35 years).

**Results:**

After multiple testing correction (*P*_CORRECTED_ < .05), rare variants were associated with basal ganglia volumes (*TREM2* and *PLCG2* effects within the putamen and pallidum, respectively). Nominal associations between *TREM2* and reduced hippocampal and thalamic volumes were also observed.

**Discussion:**

Our observations suggest that rare variants in microglia-mediated immunity pathway may contribute to the subcortical alterations observed in AD cases. These observations provide further evidence that genetic risk for AD may influence the volume of subcortical volumes and increase AD risk in early life processes.

## 1 Background

A recent exome sequencing study has revealed that risk for Alzheimer's disease (AD) is partly explained by rare (minor allele frequency <1%) single nucleotide variants within *ABI3*, *PLCG2*, and *TREM2*, genes with known roles in microglia-mediated innate immunity [1]. These variations confer amino acid substitutions, which are likely to influence protein function and or/expression. However, the neurobiological mechanisms by which these variants confer susceptibility are relatively unknown.

Preclinical studies suggest that AD is preceded by a progressive pattern of cortical/subcortical atrophy, where the earliest evidence of histopathologic changes occurs in the medial temporal lobe [2]. These alterations have been explored via magnetic resonance imaging and broadly suggest that AD risk variants may contribute to the progressive atrophy that manifests in preclinical markers such the volume (cubic millimeters) of subcortical structures such as the hippocampus and amygdala. Briefly, early work implicated the strongest known common genetic risk factor for AD (*APOE* locus) with alterations in the volume of these structures, in early and later life processes [3]. More recent work has adopted a multivariate approach to cumulatively assess the impact of all common genome-wide association studies–identified AD risk alleles [4] (as assessed via a polygenic risk scores) on brain structures such as the hippocampus, suggesting that the combined influence of common risk alleles for AD may confer risk in early life process [3], [5], [6], [7], [8], [9], [10]. Together, these neuroimaging genetic studies provide insight into the mechanisms by which common risk alleles for AD may increase risk and suggest that AD risk alleles may shape the medial temporal lobe volumetry, making them more vulnerable to atrophy in later life.

However, little is known about rare coding variants' influence on the AD-susceptible regions of the brain. The preliminary evidence suggests that comparable to the combined effects of common AD risk alleles, rare AD variants within *TREM2* are also associated with reduced hippocampal volume, in older individuals [7], [11]. Together, these studies suggest that both common and rare risk alleles for AD may confer susceptibility by common pathways. However, the impact of recently identified variants within *ABI3* and *PLCG2* on neuroimaging markers such as subcortical volume has not been assessed. On the basis of the common biological function by which these locus operate (microglia-mediated innate immunity), it is anticipated that these rare risk alleles will also contribute to the volumetric alterations that precede AD symptomology.

In the present study, the subcortical volumes of carriers of minor alleles within *ABI3*, *PLCG2*, and *TREM2* with noncarriers are compared, in a large population of young, healthy individuals. The impact of these risk alleles is explored in a young cohort to minimize the confounding impact of orthogonal, interactive environmental AD risk factors that also influence subcortical brain volumes such as age and lifestyle factors [12], [13], [14]. On the basis of the prior evidence suggesting that subcortical volume alterations may be a common mechanism by which common and rare AD risk allele may confer susceptibility [7], it is anticipated that the individuals that possess the risk alleles at these loci will have alterations in subcortical volumetry, specifically within the medial temporal lobe (hippocampus, amygdala).

## 2 Materials and methods

### 2.1 Participants

Data were drawn from the publicly available repository of the WU-Minn Young Adult Human Connectome Project; (http://www.humanconnectome.org/). The scanning protocol was approved by the Washington University in the St. Louis's Human Research Protection Office, Institional Review Board no. 201204036. No experimental activity with any involvement of human subjects took place at the author's institutions. Participants were drawn from the March 2017 public data release from the Human Connectome Project, a cross-sectional, multimodal genetic-neuroimaging cohort of young adults (N = 1206). All participants were aged from 22 to 35 years, for all inclusion/exclusion criteria, see Van Essen et al. [15]. Briefly, the study excluded individuals with a personal history of psychiatric disorder, substance abuse, neurologic or cardiovascular disease, and associated hospitalization or long-term (>12 months) pharmacologic/behavioral treatment. For a full brief of inclusion/exclusion criteria, please see Supplemental Table 1 of Van Essen et al. [15]. Participants were excluded from the current analyses if they lacked good-quality structural magnetic resonance imaging data, or had missing relevant genetic, interview/questionnaire data. Further information about the HCP kinship structure is available at http://www.humanconnectome.org/storage/app/media/documentation/s1200/HCP_S1200_Release_Reference_Manual.pdf. To control for population bias, the sample was further restricted to individuals of Caucasian descent. Each of the three variant groupings (*ABI3*, *PLCG2*, and *TREM2*) were equally represented across gender and *APOE* ɛ4 status (χ^2^ test, *P* > .1, in all cases) and did not differ in age, handedness, body mass index, education, and employment (independent sample *t* test, *P* > .1, in all cases). See Table 1 for complete description of the demographic data.

**Table 1:** Demographic details for the final sample, stratified by *ABI3, PLCG2*, and *TREM2* genotypes

N = 766	*ABI3*: rs616338 (minor allele = A)				*PLCG2*: rs72824905 (minor allele = G)				*TREM2*: rs143332484 (minor allele = T)			
	GG N = 748		AG N = 18		CC N = 743		GC N = 23		CC N = 752		TC N = 14	
Age	29.024	3.590	29.167	3.714	29.046	3.588	28.435	3.691	29.013	3.594	29.786	3.468
Gender (F/M)	394/354		10/8		394/349		10/13		394/358		10/5	
Handedness	68.242	41.390	66.944	34.816	68.082	41.345	72.391	37.865	68.351	41.034	60.714	51.882
*APOE*ɛ4 (−/+)	568/180		12/6		561/182		19/4		571/181		9/5	
Body mass index	26.229	4.928	28.910	5.374	26.298	4.980	26.113	4.039	26.287	4.963	26.553	4.500
Education	15.082	1.699	14.722	1.873	15.078	1.700	14.913	1.832	15.072	1.704	15.143	1.703
Employment	1.588	0.695	1.444	0.784	1.585	0.697	1.565	0.728	1.584	0.697	1.643	0.745

NOTE. Mean ± standard deviation. *APOE* ɛ4 (−/+) represent individuals who possessed at least one copy of the *APOE* ɛ4 allele. Handedness was assessed via the Edinburgh handedness scale. Education and employment were assessed via SSAGA_Education and SSAGA_Employment, respectively, as described in the Young Adult Human Connectome Project data dictionary: https://wiki.humanconnectome.org/display/PublicData/HCP+Data+Dictionary+Public-+Updated+for+the+1200+Subject+Release.

### 2.2 Genotyping

All Young Adult Human Connectome Project data are publicly available, including genome-wide genotype data to be distributed through the database of genotypes and phenotypes. From this data set, 1141 subjects were genotyped, and 1,580,642 single-nucleotide polymorphism (SNPs) passed initial quality control. Quality control was implemented in PLINK v1.9 [16]. Briefly, SNPs were excluded if the call rate was less than 98%, or if the χ^2^ test for Hardy-Weinberg equilibrium had a *P* value less than 1 × 10^−4^. Individuals were excluded for ambiguous sex (genotypic sex and phenotypic sex) not aligning or genotyping completeness less than 97%. The candidate variants within *ABI3* (rs616338), *PLCG2* (rs72824905), and *TREM2* (rs143332484) passed quality control. No individual in the sample possessed two copies of the minor allele at any of the three loci. Variation in the sample kinship structure was further controlled in all analysis (see Section 2.4). Individual *APOE* status was also determined by the absence/presence of a ɛ4 allele (rs7412; rs429358).

### 2.3 Data acquisition, preprocessing, and quality control

Human Connectome Project sample: Images were acquired using a customized Siemens Skyra 3-T scanner with a 32-channel head coil. For details on data acquisition and preprocessing, see Glasser et al. [17]. Subcortical and intracranial volumes (cubic millimeters) were estimated with Freesurfer v5.2 [18], which were subsequently used for the Young Adult Human Connectome Project minimal processing pipeline [17]. Seven subcortical volumes (accumbens, amygdala, caudate, globus pallidus, hippocampus, putamen, and thalamus) were averaged across hemisphere and adjusted for intracranial volume, a method previously established by recent genomic studies part of the Enhancing Neuro Imaging Genetics through Meta-Analysis consortium [19], [20]. After all complete data (N = 766) were considered and the statistical outliers for each subcortical volume were determined, the final sample size range was N = 756–765).

### 2.4 Statistical inferences

Linear mixed-effects (LME) models were estimated in R (https://www.r-project.org/) using the ‘*lmer*’ package, as a previously recommended solution for regression models with latent familial correlation structure [21]. Briefly, each of the seven corrected subcortical volumes was entered into mixed-effects models with *ABI3*, *PLCG2*, and *TREM2* as fixed effects, age, sex, *APOE* status (absence/presence of an *APOE* ɛ4 allele), handedness, body mass index, education, and employment covariates of no interest. To account for the familial structure in the sample, a sparse kinship matrix was included in each of the seven LME models using the “lme4qt” extension package [22] (see Equation 1). Subcortical volumes that were statistical outliers were removed using the interquartile range outlier labeling rule (1.5 × interquartile range (Q3–Q1)) as previously described [23]. *P* values were adjusted by corrected for the number of LME models (*P*_CORRECTED_ = .05/7). To further establish potential confounding from the kinship structure, we split the sample into “twin” and “nontwin” samples and re-estimated the effects using the “metafor” package [24].

(1) γ=Χβ+Ζυ+ε

Equation 1 is taken from [22]. When *n* equals the sample size, *X*[*n* × *p*] and *Z*[*n* × *n*] are incidence matrices and *p* is the number of fixed effects (SNPs and covariates). β[*p* × 1] is a vector of fixed effects, *n* × 1 is a vector of a random polygenic effect, and ε[*n* × 1] reflects the residual error.

## 3 Results

**Fig. 1:**
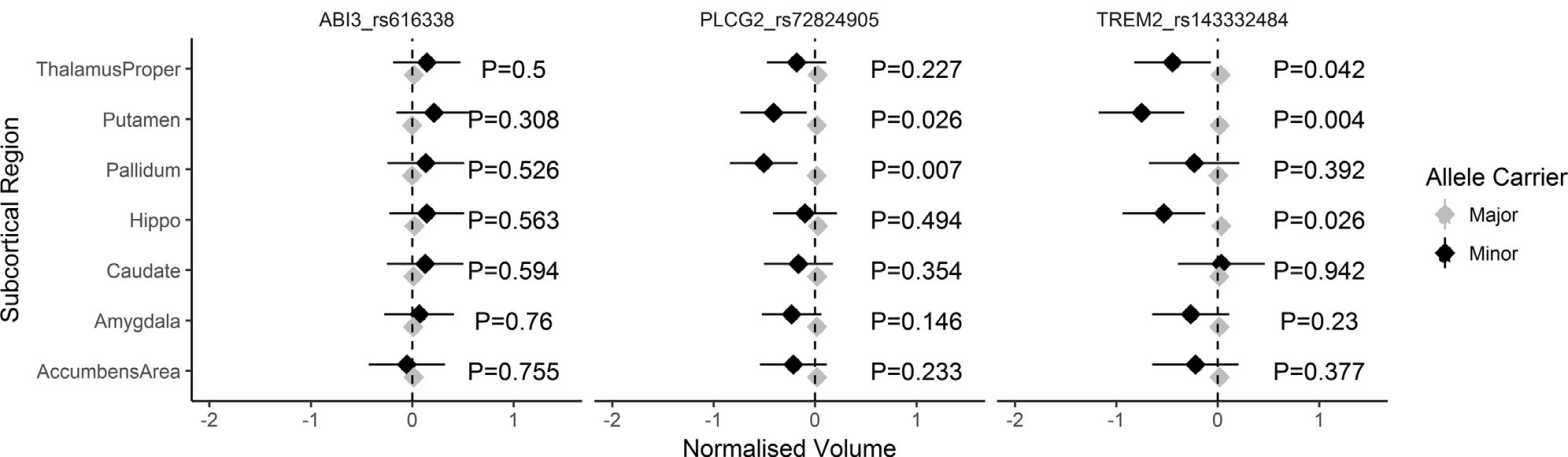
X-axis = corrected volumes (adjusted for all covariates) across seven subcortical volumes (Y-axis) for the three variants. Abbreviations: Black = minor allele; gray = major allele. Error bars represent 95% confidence intervals.

There were no associations (corrected/uncorrected) between *ABI3* variant and subcortical structures. After controlling for the multiple testing, the minor allele at the *PLCG2* locus was associated with reduced volume (cubic millimeters) in the pallidum (*P*_CORRECTED_ = .035) and the *TREM2* locus and volume in the putamen (*P*_CORRECTED_ = .028). We observed nominal associations between (1) *PLCG2* and the putamen (*P*_UNCORRECTED_ = .026) and (2) between *TREM2* and the hippocampus (*P*_UNCORRECTED_ = .026) and thalamus (*P*_UNCORRECTED_ = .042). See Table 2 and Fig. 1 for all estimated effect sizes and 95% confidence intervals.

**Table 2:** Standardized beta estimate of each variant on the seven subcortical volumes. Effect estimate reflects the impact of the minor allele at each of the three variants

Variant	Volume (mm^3^)	Standard β	Std. Error	*P*
*ABI3*_rs616338	Accumbens area	−0.06729	0.215404	.754754
	Amygdala	0.059058	0.193288	.759951
	Caudate	0.114756	0.215069	.593634
	Hippo	0.121099	0.209561	.563353
	Pallidum	0.134907	0.21283	.526165
	Putamen	0.214194	0.210183	.308164
	Thalamus proper	0.127384	0.188806	.499878
*PLCG2*_rs72824905	Accumbens area	−0.23319	0.195503	.23296
	Amygdala	−0.24926	0.171324	.145704
	Caudate	−0.18399	0.198696	.354459
	Hippo	−0.12729	0.186125	.494059
	**Pallidum**	**−0.52205**	**0.192925**	**.00681**
	Putamen	−0.42581	0.190697	.025554
	Thalamus proper	−0.20704	0.171346	.226934
*TREM2*_rs143332484	Accumbens area	−0.2366	0.267889	.377128
	Amygdala	−0.28245	0.235478	.23034
	Caudate	0.019829	0.273673	.942241
	Hippo	−0.56729	0.255265	.02626
	Pallidum	−0.23682	0.276474	.391683
	**Putamen**	**−0.76972**	**0.265246**	**.003709**
	Thalamus proper	−0.47425	0.233696	.042423

NOTE. Bold represents significant associations after multiple testing correction.

Abbreviations: β = Standardized beta estimate; Std. Error = standard error of effect estimate.

**Fig. 2:**
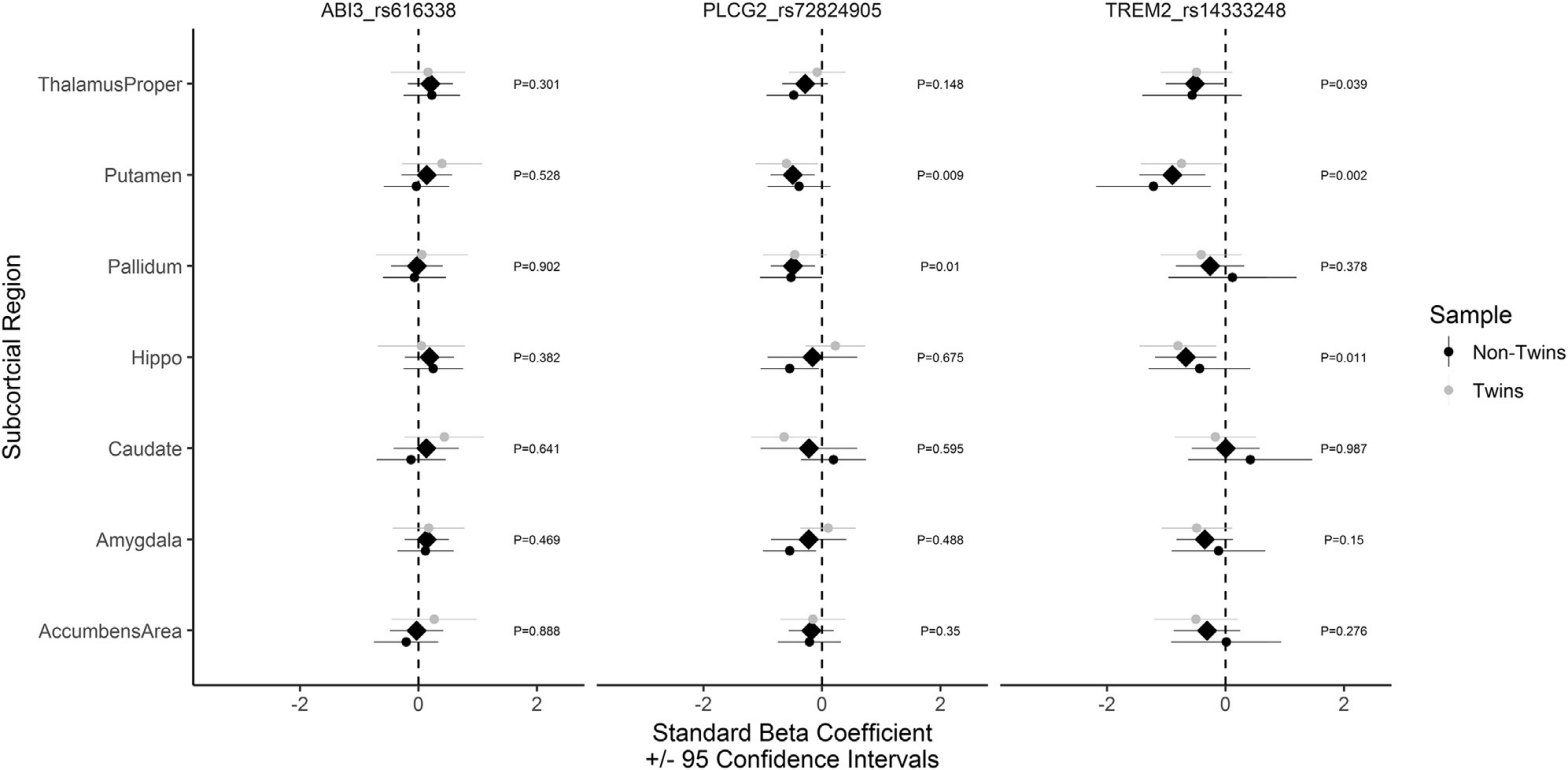
X-axis = Standardized beta coefficient (adjusted for all covariates) across seven subcortical volumes (Y-axis) for the three variants. Abbreviations: Black (circle) = twin sample; gray (circle) = nontwin sample. Black (diamond) = pooled estimate. Error bars represent 95% confidence intervals.

To further control for variation in the kinship structure, we then proceeded to split the sample into twins and nontwin groups. The pooled effects remained largely unchanged: *PLCG2*-pallidum, *P* = .01; *PLCG2*-putamen, *P* = .009; *TREM2*-hippocampus, *P* = .011; and *TREM2*-putamen, *P* = .039. See Fig. 2 for estimated effects and 95% confidence intervals.

## 4 General discussion

The association between three rare microglia-linked AD risk alleles on subcortical volumes was assessed in a large sample of young individuals. Consistent prior reports [7], [11], the rare AD-associated variation within *TREM2* was nominally associated with reduced hippocampal volume. Here, these prior observations are expanded on to show that these alterations are also present in early life process. Together, accumulating evidence now suggests that both common and rare variations that confer risk for AD may converge on pathophysiological mechanisms such as alterations in subcortical volumetry in early life [6], [8]. Furthermore, there was a negative association between the *TREM2* risk allele and volume of putamen. This adds to evidence from previous studies have also shown that common AD risk loci such as *BIN1* and *ABCA7* are also associated with alterations in putamen volumes [25]. We further observed negative associations between the *PLCG2* protective locus and pallidum/putamen volumes. The observations between *PLCG2/TREM2* and basal ganglia volume provide novel insight into mechanisms by which microglia-mediated innate immunity may confer risk for AD. It is of interest that the minor allele at *PLCG2* (protective allele) and *TREM2* (risk allele) were both associated with a reduction in putamen volume. This suggests that although putamen volume may be a common mechanism by which rare AD genetic risk is conferred, the precise molecular mechanisms that lead to opposing phenotypes should be explored. The *ABI3*, *PLCG2*, and *TREM2* transcripts have a common expression pattern in human brain cortex, with high expression in microglia cells and limited expression in neurons, oligodendrocytes, astrocytes, and endothelial cells, suggesting that future imaging genetic studies of AD-related risk would benefit from imaging measures sensitive to microglia function [26], [27], [28].

Together, these observations suggest that rare AD risk alleles may also confer susceptibility via alterations in subcortical volumes, specifically in the medial temporal lobe and basal ganglia. The AD-associated risk loci within *PLGC2* and *TREM2* confer protein changes, these variants may influence subcortical volumetry via alteration in protein function and/or expression, which have yet to be elucidated. Our observation is also supported by recent histologic evidence showing that AD genetic risk (with and without *APOE*) is associated microglia density exclusively within temporal lobe structures [29]. Innate immunity represents a significant component in the broad genetic architecture of AD [29]; however, the relationship between neuroimaging markers of AD risk and immune function remains limited. Initial imaging studies have shown that marker of inflammation such as C-reactive protein are associated with cortical volume loss [30], [31].

Our observations should be interpreted with several considerations. First, as these three variants considered in this analysis are uncommon (minor allele frequency <1%), these sample sizes for the respective minor alleles were small (N_RANGE:_ 14–23). However, as our observations are consistent with prior associations [7], [11], [32], we suggest that this work may add important insight into rare AD variants across the lifespan. Second, as the effects of these variants were assessed in a cross-sectional sample, it is unknown how variants affect brain structure across the lifespan. Furthermore, large imaging consortia projects such the lifespan development and aging projects will be instrumental in assessing the impact of common and rare AD risk alleles in early and later-life processes [33]. Finally, it is of note that the rare variants were largely contained within families. Our (A) mixed-effects models and (B) split-half analysis provide support in the findings, providing further evidence for associations between hippocampal volume and *TREM2* [7], [11]. There is also evidence that rare variation in *TREM2* may also influence basal ganglia volumes [32] and altered markers of brain health (such as cerebral blood flow/ventricular expansion) in animal models [34], [35]. However, observations between *PLCG2* and putamen volume should be interpreted with caution until replicated in independent samples. Nevertheless, the associations between the *PLCG2* and *TREM2* loci and putamen/globus pallidus broadly suggest that the volumetric reductions in basal ganglia may reflect a immunogenic mechanism of AD-linked susceptibility that manifests in early life process and a potential target for detection, prevention, and intervention.

## 5 Conclusion

This study demonstrates associations between subcortical volumes (basal ganglia) and rare AD genetic risk in locus with known functions in microglia-mediated innate immunity (*PLCG2* and *TREM2*). These observations contribute to a growing body of evidence suggesting that genetic variation (rare and common) may contribute to AD risk in early life by influencing the volume of subcortical structures. Future bioinformatics research will help to refine and uncover the principle biological gradients (such as microglia-mediated innate immunity) that underpin AD genetic risk and neuroimaging correlates [36]. Future in vivo neuroimaging measures that map immunity/microglia function in the brain will elucidate unknown mechanisms of AD risk and aid in the understanding of the pathophysiology preceding clinical symptomology.

**Research in Context**

1. Systematic review: Recent exome sequencing studies have identified rare variants in *AIB3, PLCG2*, and *TREM2* that influence risk for Alzheimer's disease (AD). However, the mechanisms by which these loci confer risk are largely unknown. This study explores the relationship between these risk variants and brain volumes (via magnetic resonance imaging) in early life process.
2. Interpretation: *TREM2* risk was associated with smaller putamen volumes. The *PLCG2* variant was further associated with reduced pallidum volume. These variants may influence subcortical volumetry at an early age, which may confer risk for AD in later life.
3. Future directions: Microglia–mediated innate immunity may be a key component of AD genetic risk that influences brain health. Future neuroimaging studies that map immunity/microglia function in the brain will elucidate unknown mechanisms of AD risk and aid in the understanding of the pathophysiology preceding clinical symptomology.
